# Slow Binocular Rivalry as a Potential Endophenotype of Schizophrenia

**DOI:** 10.3389/fnins.2018.00634

**Published:** 2018-09-12

**Authors:** Guixian Xiao, Kongliang He, Xingui Chen, Lu Wang, Xiaomeng Bai, Liling Gao, Chunyan Zhu, Kai Wang

**Affiliations:** ^1^Department of Neurology, The First Affiliated Hospital of Anhui Medical University, Hefei, China; ^2^Anhui Province Key Laboratory of Cognition and Neuropsychiatric Disorders, Hefei, China; ^3^Collaborative Innovation Center for Neuropsychiatric Disorders and Mental Health, Anhui, China; ^4^Anhui Mental Health Center, Hefei, China; ^5^Department of Medical Psychology, Anhui Medical University, Hefei, China

**Keywords:** binocular rivalry, Schizophrenia, sibling, visual endophenotype, cognitive function

## Abstract

**Objectives:** Binocular rivalry is a typical example of bistable perception that arises when two monocular images are simultaneously presented to each eye. Binocular rivalry is a heritable perceptual cognitive function that is impaired in patients with schizophrenia (SZ). Despite its potential suitability as a visual endophenotype, binocular rivalry has hardly been studied in the unaffected siblings of schizophrenia (SIB). There is also little research about whether binocular rivalry is a potential visual endophenotype between SZ and SIB.

**Methods:** In our cross-sectional study, we included 40 SZ and their unaffected SIBs, as well as 40 age- and sex-matched healthy controls (HC). All subjects underwent the binocular rivalry test, the Positive and Negative Syndrome Scale (PANSS) and a battery of cognitive neuropsychological assessments evaluating attention, memory and executive function domains.

**Results:** Our results demonstrate that the switching rate in SZ was significantly slower than in HC (*p* < 0.001), and compared to the SIB, the mean alternation rates were significantly different (*p* < 0.01). Moreover, there was a significant difference in mean switching rate between the SIB and the HC (*p* < 0.001). There was no significant correlation between the alternation rate of binocular rivalry and these cognitive tasks and the PANSS scores.

**Conclusion:** The present study shows that SZ and SIB both exhibit changes in binocular rivalry, with SIB exhibiting intermediate performance compared with that of SZ and the HC. This supports the claim that the switching rate for SZ differs from that of SIB and suggests that binocular rivalry may qualify as a visual endophenotype for SZ.

## Introduction

Binocular rivalry is a typical example of bistable perception that was first mentioned by Porta in the 16th century and later by DuTour in more detail (Blake and Logothetis, 2002; Baker et al., 2015; Chang et al., 2016). When two monocular images are simultaneously presented to each eye, the brain cannot form a single stable percept, leading to the alternation of percepts. The observer perceives one image at a time, and perceptual switches occur stochastically on the order of seconds. Binocular rivalry has been widely investigated in the fields of cognitive and visual science and is linked to the superior parietal lobe. In recent years, an increasing number of studies have begun to explore the phenomenon of binocular rivalry in the context of psychiatric diseases (Miller et al., 2003; Jia et al., 2015).

Schizophrenia is a phenotypically and genetically heterogeneous neuropsychiatric disorder with general cognitive impairments (Gottesman and Shields, 1973; Kim and Kim, 2017). In schizophrenia, there are two core symptom types: positive and negative. Cognitive deficits have been recognized as the core features of schizophrenia. Nearly a century ago, Gallhofer first proposed cognitive dysfunction as a third core symptom of schizophrenia; cognitivedys function is linked to both positive symptoms and negative symptoms and is exhibited by most patients with schizophrenia (Gallhofer, 1997; Misiak et al., 2017). MRI studies have shown abnormalities in schizophrenics in many brain regions, including the prefrontal cortex, parietal lobe, temporal lobe, hippocampus, occipital lobe, and cerebellum (Knapen et al., 2011; Liu X. et al., 2016). Studies have also shown that multiple brain regions in schizophrenics exhibit structural and functional abnormalities, including the parietal and temporal lobes and the cerebellum, suggesting that the pathophysiology of schizophrenia involves the disruption of a variety of neural networks, leading to cognitive impairment (Chang et al., 2014, 2016).

Studies have shown that schizophrenia exhibits a degree of heritability: relatives of patients have a prevalence of schizophrenia several times higher than that of the general population, and the closer the blood relationship to a schizophrenic, the higher the prevalence. In some family surveys, a high prevalence of schizophrenia was found to be associated with a high prevalence of family history, especially among immediate family members. Thirty years ago, Gottesman and Shields (Gottesman and Shields, 1973) described “endophenotypes” as internal phenotypes discoverable by “biochemical test or microscopic examination” (Todd and Gould, 2003). Endophenotypes are behaviors or characteristics that are intermediate between genotype and a phenotype of interest. More specifically, an endophenotype is defined as a hereditary quantitative trait that is considered to be intermediate between the disease phenotype and its underlying biological process (Reus and Freimer, 1997). Common assessment indicators used for endophenotype analysis include biochemistry, neuroimaging, neuroendocrinology, endocrinology, and neuropsychological approaches. Among them, the measurement of neurocognitive function is widely regarded as a valuable neurocognitive endophenotype due to its demonstrated reliability and stability. Patients with schizophrenia and their unaffected siblings exhibit abnormal activation of certain brain regions (Li et al., 2012). Previous studies have shown that schizophrenia may be characterized by abnormal lateralization (Guo et al., 2014) during development. This study also found that patients and their unaffected siblings share decreases in the volume of the left middle temporal back gray matter, presenting a potential inherent phenotype of schizophrenia (van der Velde et al., 2015; Pergola et al., 2017).

Neurological cognitive deficits have been identified as potential intrinsicphenotypes of schizophrenia. An extensive search for candidate endophenotypes has been conducted for some psychiatric disorders, including obsessive–compulsive disorder (Zhang et al., 2015), bipolar disorder (Bora et al., 2009), autism (Delorme et al., 2007), and attention deficit hyperactivity disorder (Albrecht et al., 2008). Most searches for endophenotype markers of SZ have found links to brain network connectivity (Chahine et al., 2017), gene expression (DiLalla et al., 2017), and psychophysiological organization (Liu M. et al., 2016). However, to date, few studies of SZ endophenotype have focused on neurocognitive functions. Prior studies have found deficits in executive function (Trail Making Test) (Aydin et al., 2017), memory (working memory (de Leeuw et al., 2013) and declarative memory (Trandafir et al., 2006) and attention (Massuda et al., 2013). However, the basic nature of the visual characteristics of siblings is unclear, especially in terms of binocular competition.

Binocular rivalry is widely studied in the fields of cognitive and visual science, and its function has been linked to the superior parietal lobe (Baker et al., 2015). Interestingly, the structural and functional abnormalities present in schizophrenia involve the frontoparietal cortex (Lumer et al., 1998). Studies have shown that TMS application in the anterior parietal lobe causes binocular rivalry and bistable perception alternations to accelerate (Baker et al., 2015). However, the rate of bistable perception is reduced in patients with mental disorders, including BD and depression (Miller et al., 2003; Jia et al., 2015), compared to healthy controls. Study has demonstrated that binocular rivalry is slower in first-degree relatives of SZ (Wright et al., 2003). Nevertheless, there has been few study of binocular rivalry in schizophrenics and their siblings now. A genetic epidemiological survey revealed that the prevalence of the disease among relatives was higher than in the general population (Miller et al., 2010; Shannon et al., 2011), and the closer the blood relationship, the higher the risk of disease. Thus, in this study, we examine whether binocular rivalry is a visual endophenotype for SZ.

## Materials and methods

### Participants and clinical diagnosis

Participants consisted of 40 healthy controls (HC) (ranging from 18 to 43 years of age;18 males and 22 females), 40 patients with schizophrenia (SZ) (ranging from 17 to 48 years of age; 15 males and 25 females) and the unaffected siblings (SIB) of schizophrenia patients (ranging from 18 to 48 years of age; 15 males and 25 females).Patients with schizophrenia were diagnosed by at least two psychiatrists, and all were recruited from Department of Psychiatry outpatient clinics or the inpatient department at the Fourth People's Hospital in Hefei. Healthy controls, recruited from the nearby community, were matched with the patients in age (18–43, y = 26.39 ± 6.23) and gender. All participants met our enrollment and exclusion criteria. All healthy controls were free of mental disorders and had no symptoms of mood disorders. All participants' eyesight was normal or normal after correction, with no color blindness or other visual impairments present. All participants provided informed consent and were compensated for their participation, as approved by the Institutional Review Board of Anhui Medical University. All participants were naive to the purpose of the study and did not consume alcohol or coffee prior to the test (George, 1936).

### Neuropsychological background test

A neurological test consisting of standardized tests was used to investigate the participants' basic cognitive conditions, including anxiety and depressive symptoms in their daily lives, and was conducted by skilled psychologists and psychiatrists. The Montreal Cognitive Assessment test (MoCA Test) was used to assess general cognitive function (scores > 24 points were included in the study). The Hamilton Depression Rating Scale (HAMD) and the Hamilton Anxiety Rating Scale (HAMA) were used to assess participants for anxiety and depressive symptoms, respectively (scores > 8 points were excluded in the study). The Positive and Negative Syndrome Scale (PANSS) was used to assess the current severity of the patients' symptoms. The Verbal Fluency test(VFT), which evaluates general frontal lobe executive function, requires the participant to list as many words as possible within a minute. The Stroop test(color, word, interference), and Trail Making Test(A, B) are designed to evaluate common execution functions, and the Digit Span test(forward, backward) portion of the Wechsler Adult Intelligence Scale was used to assess attention.

### Binocular rivalry test

#### Stimuli

**Figure 1:**
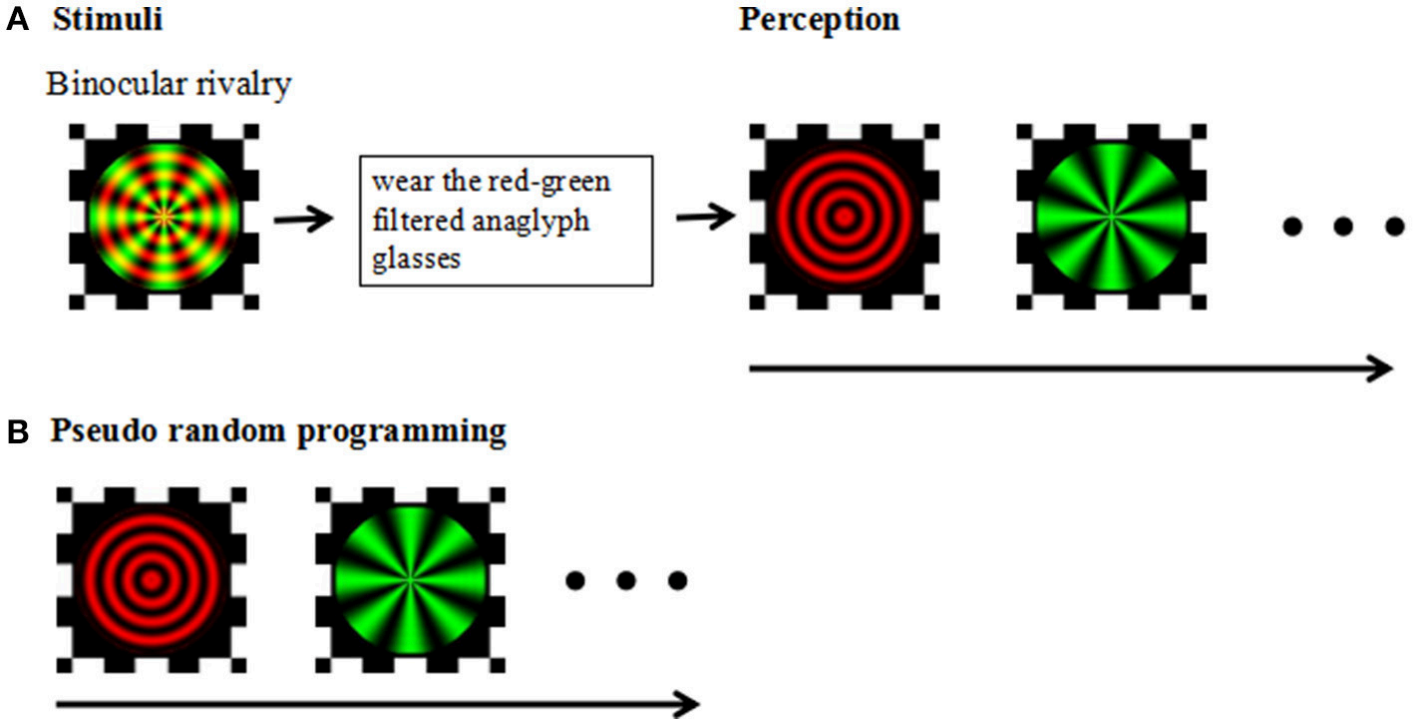
**(A)** Stimuli: Experimental design and stimuli. Perception: Following a brief practice session using red-green filtered anaglyph glasses, participants viewed red-green circular and radial gratings designed to produce stochastic alternation in visual perception between the two images (i.e., binocular rivalry). **(B)** Pseudorandom programming was used to ensure the participants' perceived task completion.

The experiment was carried out in a dark and quiet environment. All aspects of the experiment were controlled by a Lenovo ThinkPad computer running MATLAB and the Psychophysics Toolbox (Brainard, 1997; Pelli, 1997). The visual stimuli were created in Matlab using the Psych toolbox; stimuli were displayed on a Lenovo laptop with a 14-inch monitor (1366 × 768 pixels; refreshment rate, 48 Hz) and were viewed at a distance of 75 cm from the screen, yielding a viewing angle of 1.5°for the stimuli. During the experiment we asked the participants to keep their heads fixed so as not to affect the distance from the computer screen. The stimulus was a composite image consisting of red concentric circles and green radial gratings (Figure 1).

#### Procedure

The test consisted of two parts: a practice session and the binocular rivalry test. Before beginning the binocular competition test, participants were required to complete an exercise to ensure that the test was understood and that the correct response was made during the test. Practice did not require the use of red-green anaglyph glasses (a red filter over the left eye and green filter over the right eye), and the stimulus was gradually changed from red radial concentric circles to green radial gratings. After the practice session, the participants were instructed to view the superimposed red/green stimuli on the monitor through red-green anaglyph glasses and to press the space key to start the test. Participants viewed the test image and pressed the corresponding button each time they sensed that the image changed; participants were instructed to press the right arrow key of the keyboard when the image appeared to be a green radial grating and to press the left arrow key of the keyboard when the image appeared to be a red round grating. The test phase consisted of three blocks, each consisting of three 30 s tests performed with 15 s rest intervals, with one pseudorandom programming test to ensure the participants' perceived task completion. In the pseudorandom programming test, sequential stimulation of the horizontal or vertical moving gratings used in the binocular rivalry test were preset for both eyes. Each session was monitored to ensure compliance with the task.

### Statistical analyses

All demographic and clinical data were all analyzed using SPSS 17.0. In the this study, the population characteristics of the three groups and the neuropsychological background test score were in accordance with the normal distribution data. We used variance analysis (ANOVA) for analysis. Non-parametric tests were used to analyze the data for the three groups does not meet the normal distribution. Pearson correlation analysis was performed to investigate the relationship between the participants in PANSS and the switching rate. The first block was considered at raining block and was removed before analysis. The switching rate (Hz) was calculated by dividing the number of perceived switches by the total competition time. We also calculated the average accuracy of the capture test to assess the task performance of each group. The significance level was set as *p* < 0.05 (2-tailed). The data were presented as the mean ± standard deviation.

## Results

### Neuropsychological tests and clinical characteristics

Patients with schizophrenia, their unaffected siblings, and unrelated healthy controls were comparable in age, sex, and years of education. The demographic data for the participants and the clinical characteristics of the patients are shown in Table 1. One-way ANOVA analysis of variance confirmed that the schizophrenia group did not significantly differ from the two control groups (unaffected siblings and HC) in age, education, HAMA, and HAMD. Bonferroni *post-hoc* tests showed that there were significant differences among the three groups in some neuropsychological tests, such as the Stroop test [Color test [*F*_(2, 120)_ = 32.046], *p* < 0.001; word test [*F*_(2, 120)_ = 35.028, *p* < 0.001; interference test [*F*_(2, 120)_ = 28.291, *p* < 0.001]]; digit span[forward[*F*_(2, 120)_ = 6.969], *p* = 0.001; backword [*F*_(2, 120)_ = 13.236], *p* < 0.001];verbal fluency [*F*_(2, 120)_ = 11.638, *p* = 0.001]; trial making test [A [*F*_(2, 120)_ = 36.814, *p* < 0.001] and B [*F*_(2, 120)_ = 57.782, *p* < 0.001]]; and MoCA [*F*_(2, 120)_ = 36.293, *p* < 0.001] (see Table 1).

**Table 1:** The demographic and clinical characteristics and neuropsychological test results of the patient, unaffected sibling, and healthy control groups in the cross-sectional study.

**Variable**	**Schizophrenia** **patients** **(*N* = 40)**	**Unaffected** **siblings** **(*N* = 40)**	**Healthy controls** **(*N* = 40)**	**F/χ^2^**	***p*-value**
**DEMOGRAPHIC**					
Gender (M/F)	15/25	15/25	18/22	0.070	0.966
Age (years)	25.50 (5.98)	26.93 (6.65)	26.39 (6.23)	0.620	0.540
Education (years)	11.95 (2.65)	13.13 (2.94)	13.20 (3.04)	2.370	0.098
**CLINICAL CHARACTERISTICS**					
Total PANSS	47.59 (10.60)	NA	NA	NA	NA
PANSS Positive	11.08 (4.54)	NA	NA	NA	NA
PANSS Negative	10.69 (3.56)	NA	NA	NA	NA
Illness duration (years)	4.61 (3.81)	NA	NA	NA	NA
Risperidone equivalent (mg)	5.91 (1.68)	NA	NA	NA	NA
**NEUROPSYCHOLOGICAL TESTS**					
HAMA	2.38 (1.15)	2.13 (0.85)	2.60 (0.90)	2.373	0.098
HAMD	2.38 (1.37)	2.55 (0.85)	2.35 (1.15)	0.365	0.695
MoCA	24.58 (3.04)^a^, ^c^	26.83 (2.12)^b^	28.75 (0.84)	36.293	< 0.001
VFT	17.58 (3.26)^a^, ^c^	20.05 (4.83)	22.18 (4.55)	11.638	< 0.001
**ATTENTION**					
Digit span test(forward)	7.43 (0.90)^a^	7.75 (0.49)	7.88 (0.52)	6.969	0.001
Digit span test(backward)	5.10 (1.08)^a^, ^c^	5.90 (0.87)	6.13 (1.16)	13.236	< 0.001
**EXECUTIVE FUNCTION**					
Stroop Color test	19.12 (5.78)^a^, ^c^	14.22 (2.69)^b^	12.36 (2.22)	32.046	< 0.001
Stroop Word test	24.07 (8.00)^a^, ^c^	16.39 (3.26)^b^	14.90 (2.85)	35.028	< 0.001
Stroop Interference test	35.55 (11.56)^a^, ^c^	25.41 (5.99)^b^	21.93 (6.56)	28.291	< 0.001
Trail Making A	70.26 (28.38)^a^, ^c^	45.98 (16.54)^b^	32.97 (9.41)	36.814	< 0.001
Trail Making B	135.89 (39.81)^a^, ^c^	86.95 (27.17)^b^	68.25 (14.50)	57.782	< 0.001

M, male; F, female; PANSS, Positive and Negative Syndrome Scale; NA, not applicable; HAMA, Hamilton Anxiety Rating Scale; HAMD, Hamilton Depression Rating Scale; MoCA, Montreal Cognitive Assessment Test; VFT, Verbal Fluency Test.

a Schizophrenic patients and healthy controls differ significantly (P < 0.05).

b Unaffected siblings and healthy controls differ significantly (P < 0.05).

c *Schizophrenic patients and unaffected siblings differ significantly (P < 0.05)*.

### Reliability of the binocular rivalry test

To ensure the reliability of the test, trials with accuracy rates less than 90% were excluded from the study. All participants experienced genuine binocular rivalry, which was supported by the data observed. There was no significant difference among the groups in the accuracy rates of the catch trials [the accuracy rates of the groups during catch trials were all above 90% (SZ: 92.50 ± 0.14; SIB: 96.25 ± 0.07; HC: 99.167 ± 0.04)].

### Mean alternation rates of binocular rivalry

**Figure 2:**
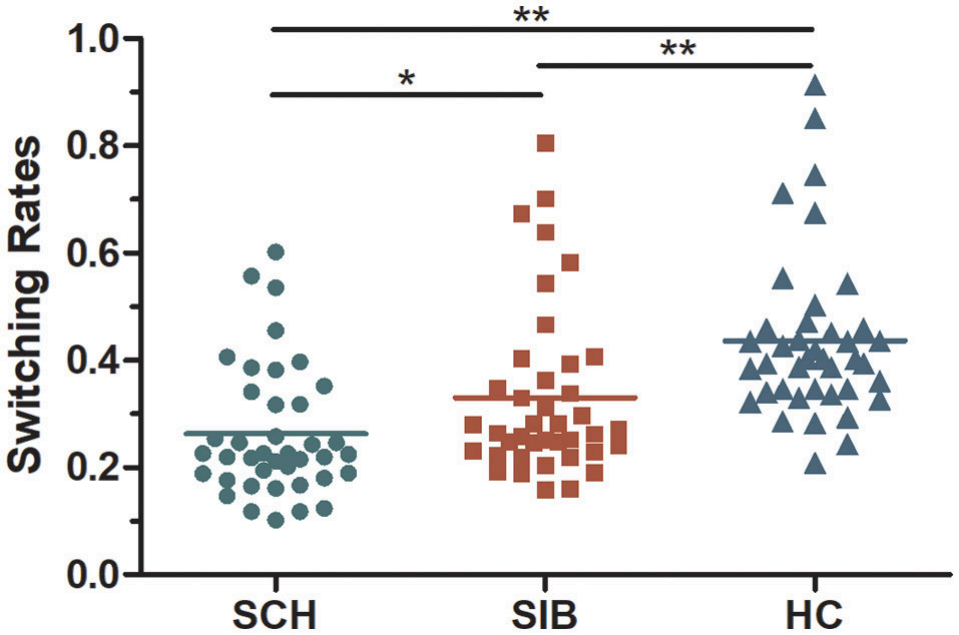
A scatter diagram of rivalry alternation rates for all participants included in the present study (mean values for each group are represented as dotted lines).

The mean switching rates of binocular rivalry for SZ, SIB and HC were 0.26, 0.33, and 0.44 Hz, respectively (Figure 2). It has shown, the dynamic binocular rivalry, especially the duration of the distribution of dominance durations or switching rates, can be well described by a gamma function. The data of this experiment also conforms to the gamma distribution (Levelt, 1965; Blake et al., 1971; Logothetis et al., 1996; Blake and Logothetis, 2002; Miller et al., 2003). Because the data of binocular rivalry did not meet the normal distribution, the Kruskal-Wallis test and Mann-Whitney tests were cited to analyze the data. K-W test of variance confirmed that the results of mean alternation rates differed significantly among the three groups [mean [χ2_(2, 120)_ = 14.468, *p* < 0.001]]. Meanwhile, M-W tests of the groups showed that the switching rate in schizophrenia patients was significantly slower than that of the controls [mean(*p* < 0.001)]; compared to the unaffected siblings, the mean switching rate differed significantly [mean (*p* = 0.039)]. Moreover, there was a significant difference in mean switching rate between the unaffected siblings and the healthy controls [mean (*p* = 0.002)] (Figure 2).

### Correlation between PANSS score, neuropsychological test score and accuracy rate with changed proportion of rivalry alternation rates in patients with schizophrenia

We performed Pearson's correlation analyses to investigate whether the slow binocular switching rates observed in schizophrenia patients and unaffected siblings were correlated with the results of the neuropsychological test. We observed no significant correlations between the binocular switching rates and PANSS scores or other neuropsychological test scores between the three groups. And Pearson's correlation analyses shows that there were no significant correlations between the binocular switching rates and accuracy rate within the three groups (SZ: *r* = −0.65, *p* = 0.69; SIB: *r* = 0.091, *p* = 0.578; HC: *r* = 0.111, *p* = 0.494).

### Power of discrimination

#### Schizophrenia patients vs. healthy controls

**Figure 3:**
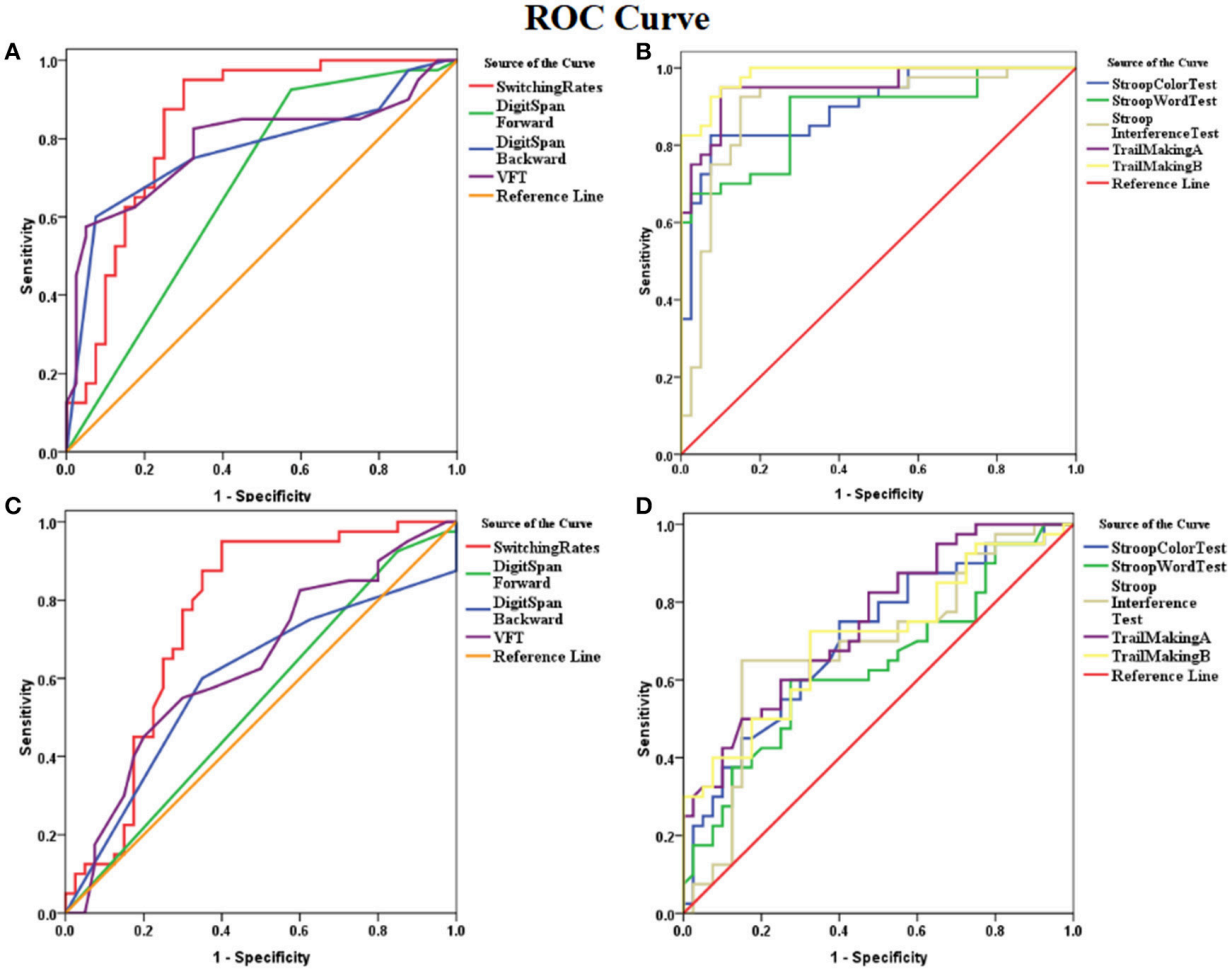
ROC (receiver operating characteristics) curves for the binocular rivalry, digit span (forward, backward), the VFT, Stroop test (color, word, interference), and Trail Making test **(A,B)**. **(A)** ROC curves for the binocular rivalry, digit span (forward, backward), and the VFT between SZ and HC. **(B)** The ROC curves for Stroop test (color, word, interference), and Trail Making test **(A, B)** between SZ and HC. **(C)** ROC curves for the binocular rivalry, digit span (forward, backward), and the VFT between SIB and HC. **(D)** The ROC curves for Stroop test (color, word, interference), and Trail Making test **(A, B)** between SIB and HC. The x-axis indicates the error of the second kind (100%-specificity). The y-axis indicates sensitivity. The area under the curve (AUC) shows the discriminative power between the two groups. The diagonal from (0,0) to (100,100) with AUC = 0.5 indicates a total lack of discriminative power.

ROC analysis showed that the binocular rivalry differentiates with other tests between patients and controls were not significant. The AUCs for the binocular rivalry, digit span (forward, backward), the VFT, Stroop test (color, word, interference), and Trail Making test (A, B) were: 0.842, 0.673, 0.770, 0.785, 0.902, 0.880, 0.900, 0.951, and 0.983, respectively (see Figures 3A, B). An AUC of 0.5 indicates the test has no discriminative power. The binocular rivalry test yields a higher discriminative power than digit span (forward, backward) and the VFT, but not Stroop test (color, word, interference) and Trail Making test (A, B). AUC values are given in the Table 2.

**Table 2:** AUC values for the binocular rivalry, digit span (forward, backward), the VFT, Stroop test (color, word, interference), and Trail Making test (A, B) of the patient, unaffected sibling, and healthy control groups in the cross-sectional study.

**Variable**	**SZ vs. HC**		**SIB vs. HC**	
	**Area**	***p*-value**	**Area**	***p*-value**
Switching Rates	0.842	0.000	0.757	0.000
Digit span (forward)	0.673	0.008	0.536	0.577
Digit span (backward)	0.770	0.000	0.595	0.142
VFT	0.785	0.000	0.638	0.034
Stroop Color test	0.902	0.000	0.706	0.002
Stroop Word test	0.880	0.000	0.636	0.036
Stroop Interference test	0.900	0.000	0.688	0.004
Trail Making A	0.951	0.000	0.747	0.000
Trail Making B	0.983	0.000	0.706	0.001

#### Siblings vs. healthy controls

ROC analysis showed that the binocular rivalry differentiates with other tests between siblings and controls were not significant. The AUCs for the binocular rivalry, digit span (forward, backward), the VFT, Stroop test (color, word, interference), and Trail Making test (A, B) were: 0.757, 0.536, 0.595, 0.638, 0.706, 0.636, 0.688, 0.747, and 0.706, respectively (see Figures 3C, D). The binocular rivalry test yields the highest discriminative power. AUC values are given in the Table 2.

## Discussion

The current results are consistent with the results of Robert Fox, who found that the duration of binocular rivalry in patients with schizophrenia is longer than in healthy controls (Fox, 1965). We also found differences between the patients and their unaffected siblings and healthy controls. There is evidence suggesting that schizophrenic patients, their unaffected siblings and their first-degree relatives exhibit cognitive changes (executive function, working memory and verbal memory deficits are the most prominent endophenotype of SZ) (Kremen et al., 2007; Nehra et al., 2016). However, to date, there have been few studies demonstrating differences in binocular rivalry between patients with schizophrenia and their unaffected siblings. Our results demonstrate that the switching rate in SZ was significantly slower than in HC, and compared to the unaffected SIB, the mean alternation rates differed significantly. Most importantly, there was a significant difference in mean switching rate between the unaffected SIB and the HC. Together, these findings suggest neurobiological differences between these groups and indicate that binocular rivalry might be a visual endophenotype in schizophrenia.

The differences between SZ and their unaffected SIB and HC cannot be explained by medication effects. The reason is still unknown now. Binocular rivalry is associated with certain brain regions, including the superior parietal lobule (SPL), intraparietal sulcus (IPS), frontoparietal cortex, dorsal lateral prefrontal cortex (DLPFC), and temporal parietal junction (TPJ) (Knapen et al., 2011; Weilnhammer et al., 2013). Functional magnetic resonance imaging (fMRI) studies have shown that unaffected siblings of schizophrenia patients exhibited slowed dynamics in the SPL—the brain region typically associated with the visual perception that normally shows decreases inactivation during binocular rivalry cognitive processes (Lyu et al., 2015)—compared to healthy controls. There may be a significant amount of white matter overlap between SZ patients and their unaffected SIB in select brain regions. Previous studies used probabilistic tractography to detect white matter fibers between right-hemispheric areas that showed event-related f MRI signal changes time-locked to reported perceptual alternations during rivalry viewing. Most of these functionally defined areas were linked by probabilistic fiber tracts, some of which followed long-distance connections such as the inferior occipitofrontal fasciculus. Corresponding anatomical pathways might mediate communication within the functional network associated with changes in conscious perception during binocular rivalry (Wilcke et al., 2009). Furthermore, studies have shown that right partial TMS shortens dominance duration in tests of binocular rivalry (Carmel et al., 2010; Kanai et al., 2011), underscoring the complex role played by the SPL in maintaining a unified perceptual experience in the face of perceptual ambiguity. According to Daniel H. Baker's study (Baker et al., 2015), SPL exhibited robust connectivity with regions of primary sensorimotor cortex. Furthermore, binocular rivalry involves several levels of processing, while the occurrence of binocular rivalry depends on local and low-level competition (Lee et al., 2007).

Studies have proposed standards for identifying markers in psychiatric genetics and these standards have been applied to the internal phenotype. Its contents include: disease-related, heritability, co-segregation, state-independent (expressed in individuals regardless of the disease is active or not), and family members are highly motivated (Gershon, 1986; Leboyer et al., 1998; Chkonia et al., 2010). Studies have shown that the slow BR does exist in both depression and bipolar disorder (Jia et al., 2015). And slow BR represents a novel candidate endophenotype for BD (Ngo et al., 2011). Our findings suggest that compared to their unaffected siblings, the switching rate of SZ was significantly slower, indicating a difference in heritability. Considering the heterogeneity of schizophrenia, there may be a potential genetic explanation about binocular rivalry. The unaffected SIB of psychosis-like performance might have psychosis-like qualities. Genetic studies of hundreds of pairs of twins have shown that there is a substantial genetic contribution to individual variation in binocular rivalry rate (Miller et al., 2010; Shannon et al., 2011). The results show that there is a genetic gradient between SZ, the unaffected SIB, and the HC in regards to binocular rivalry. However, the current study assess the bistable perception and showed that change in dissociation of binocular rivalry was an appropriate potential visual endophenotype for schizophrenia.

Due to some restrictions, the study raises some concerns. This is a cross-sectional study of neuropsychology. Since patients and their siblings are mostly grown up in the same environment, it is unclear whether the sibling's defects are caused by a similar environment. We also should have evaluated the binocular rivalry in SZ patients who had not taken antipsychotic medications. Moreover, a larger sample size is needed. In explaining our results, we should consider the limitations of the method besides. Binocular rivalry is unlikely to result from a single process but from an assembly of perceptual processes underlying instigation of rivalry, promotion of dominance and implementation of suppression. According to previous research, competition involves neural competition at multiple levels of the visual pathway (Blake and Logothetis, 2002; Kanai et al., 2011). We reviewed recent human neuroimaging and psychophysical studies, revealing the paradoxical nature of rivalry. Studies have demonstrated patients with schizophrenia displayed impaired visual contrast sensitivity, which was associated with sensory integration deficits. There are three types of cone cells, which are sensitive to red, green, and blue. Red concentric circles and green radial gratings were used in our experiments. Whether the slower switching rate is caused by contrast sensitivity damage or a rivalry deficit, it still needs further exploration. Maybe in the future we should add some experiments to verify. In our study, then we may consider MRI images of people with schizophrenia and their unaffected siblings and explore the functional connectivity of the upper lobules between them.

In summary, our results show that patients who have schizophrenia and their unaffected siblings both exhibit impairments in visual perception and cognition, and both have lower binocular switching rates than healthy controls. In addition, there is a significant difference between the patients with schizophrenia and their unaffected siblings. Our study demonstrated that changes in binocular rivalry may represent a visual endophenotype for schizophrenia.

## Ethics statement

The study was approved by the Medical Ethics Committee of Anhui Medical University. Written informed signed consent was provided by each participant before being included in the study.

## Author contributions

All these authors contributed to this work GX, KH, XC, LW, XB, LG, CZ, and KW. GX and KH wrote the first draft of the manuscript. All the authors have personally reviewed the manuscript and gave final approval of the version attached.

### Conflict of interest statement

The authors declare that the research was conducted in the absence of any commercial or financial relationships that could be construed as a potential conflict of interest.
